# Supplementary material for: Eg5 activity and density-driven bundling organize the human metaphase mitotic spindle independently of spindle bipolarity
Source: bioRxiv. 2026 Jan 16:2026.01.16.699769. Preprint. [Version 1] doi: 10.64898/2026.01.16.699769 (PMC12871229; doi:10.64898/2026.01.16.699769)
Supplement: 1 [file NIHPP2026.01.16.699769V1-supplement-1.pdf]

bioRxiv preprint doi: https://doi.org/10.64898/2026.01.16.699769; this version posted January 16, 2026. The copyright holder for this preprint (which was not certified by peer review) is the author/funder, who has granted bioRxiv a license to display the preprint in perpetuity. It is made available under aCC-BY 4.0 International license.

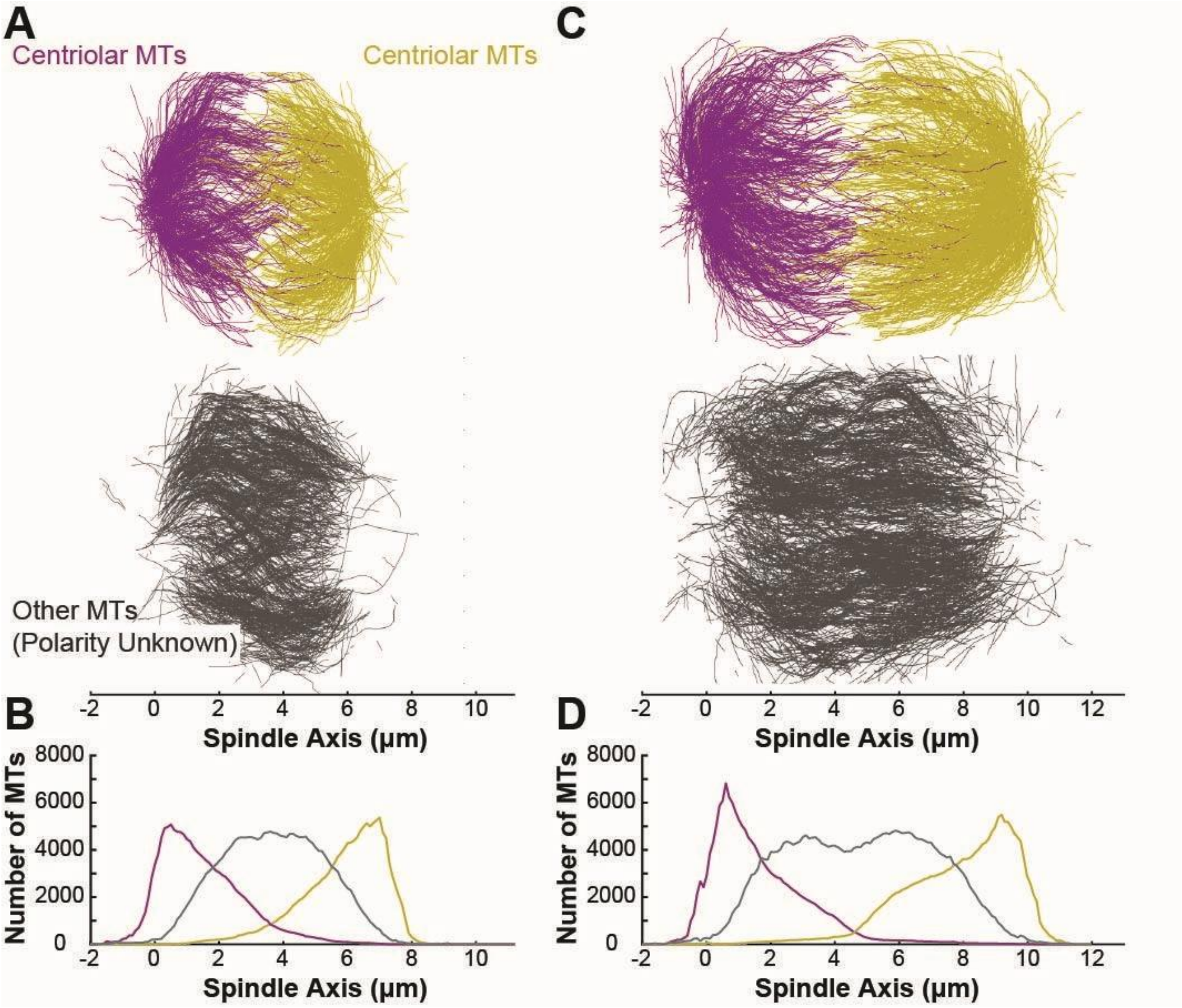

Figure 1 - supplement 1: Polarity assignment in resin-embedded serial-section electron tomography reconstructions of HeLa spindles. A) Reconstruction of microtubule trajectories from serial-section, resin-embedded electron tomography of a metaphase HeLa spindle. Microtubules are categorized by distance from either pole. Top: microtubules within 2 μm of the left (purple) or right (yellow) pole. Bottom: microtubules >2 μm from either pole (gray). B) Density of microtubules within 2 μm of the left pole (purple), within 2 μm of the right pole (yellow), and more than 2 μm away from either pole (gray).

bioRxiv preprint doi: https://doi.org/10.64898/2026.01.16.699769; this version posted January 16, 2026. The copyright holder for this preprint (which was not certified by peer review) is the author/funder, who has granted bioRxiv a license to display the preprint in perpetuity. It is made available under aCC-BY 4.0 International license.

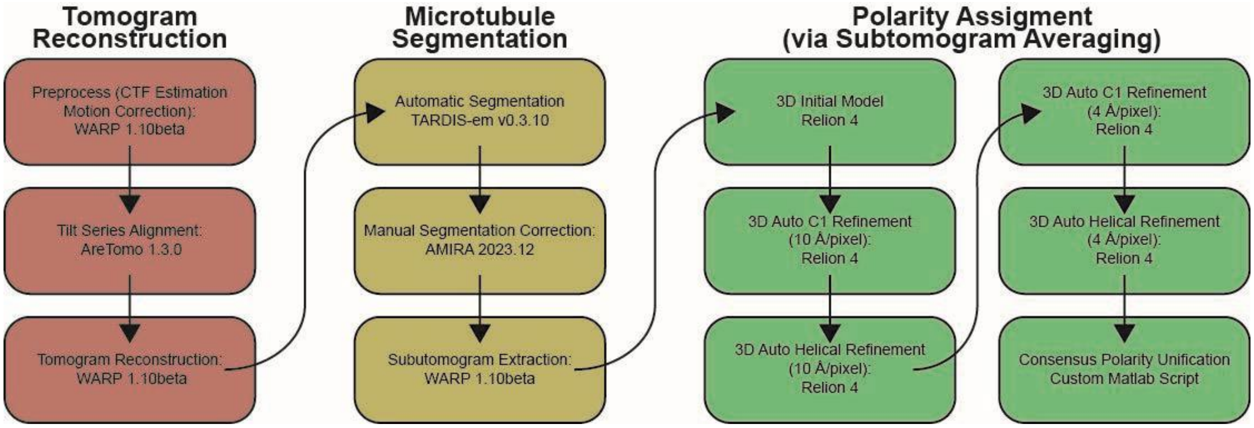

Figure 2 - supplément 1: Microtubule polarity assignment pipeline

bioRxiv preprint doi: https://doi.org/10.64898/2026.01.16.699769; this version posted January 16, 2026. The copyright holder for this preprint (which was not certified by peer review) is the author/funder, who has granted bioRxiv a license to display the preprint in perpetuity. It is made available under aCC-BY 4.0 International license.

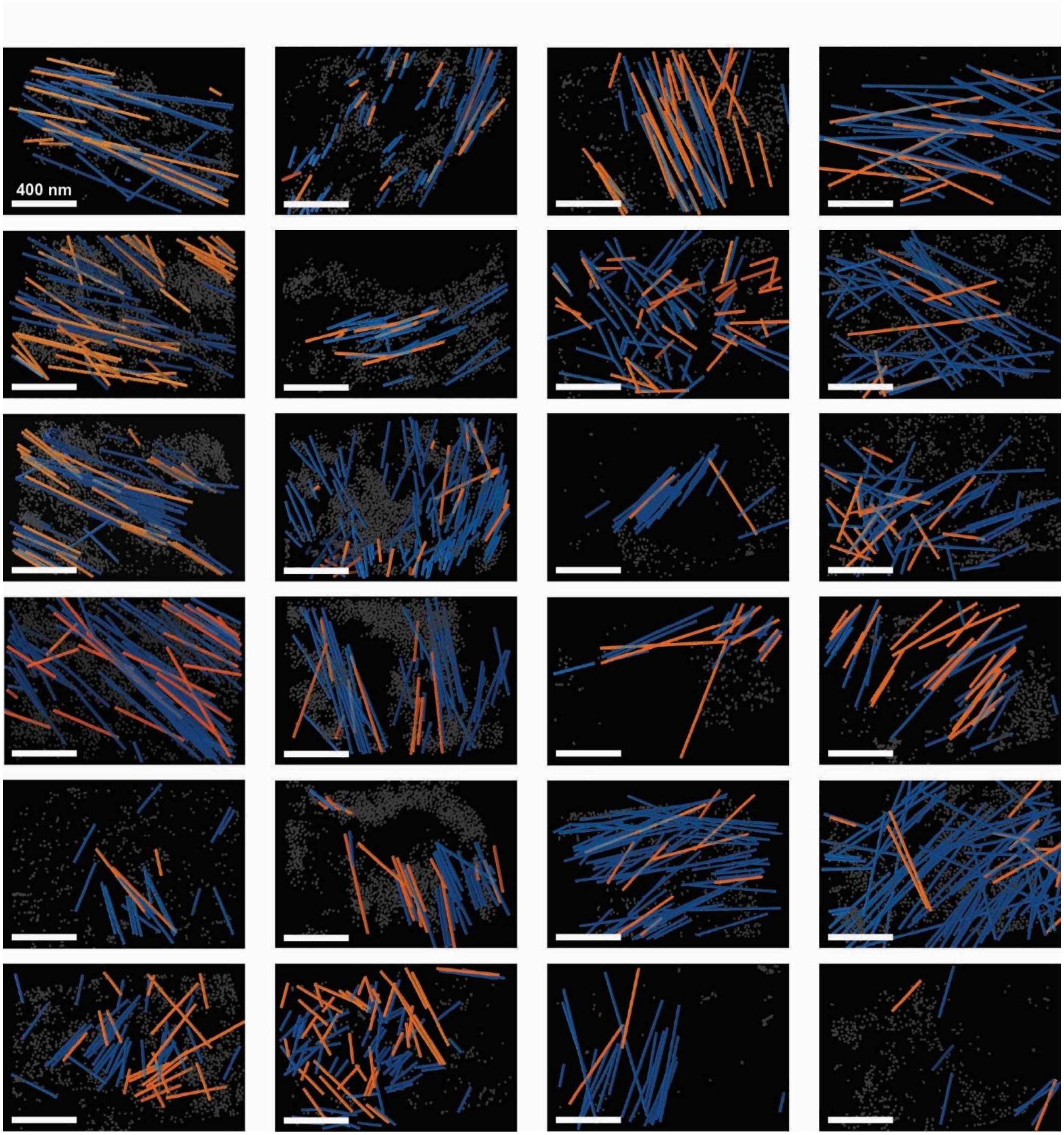

999
1000 **Figure 2 – supplement 2: Tomographic reconstructions of metaphase spindles.**
1001 Blue/orange: microtubules colored by polarity. Gray: ribosomes.

bioRxiv preprint doi: https://doi.org/10.64898/2026.01.16.699769; this version posted January 16, 2026. The copyright holder for this preprint (which was not certified by peer review) is the author/funder, who has granted bioRxiv a license to display the preprint in perpetuity. It is made available under aCC-BY 4.0 International license.

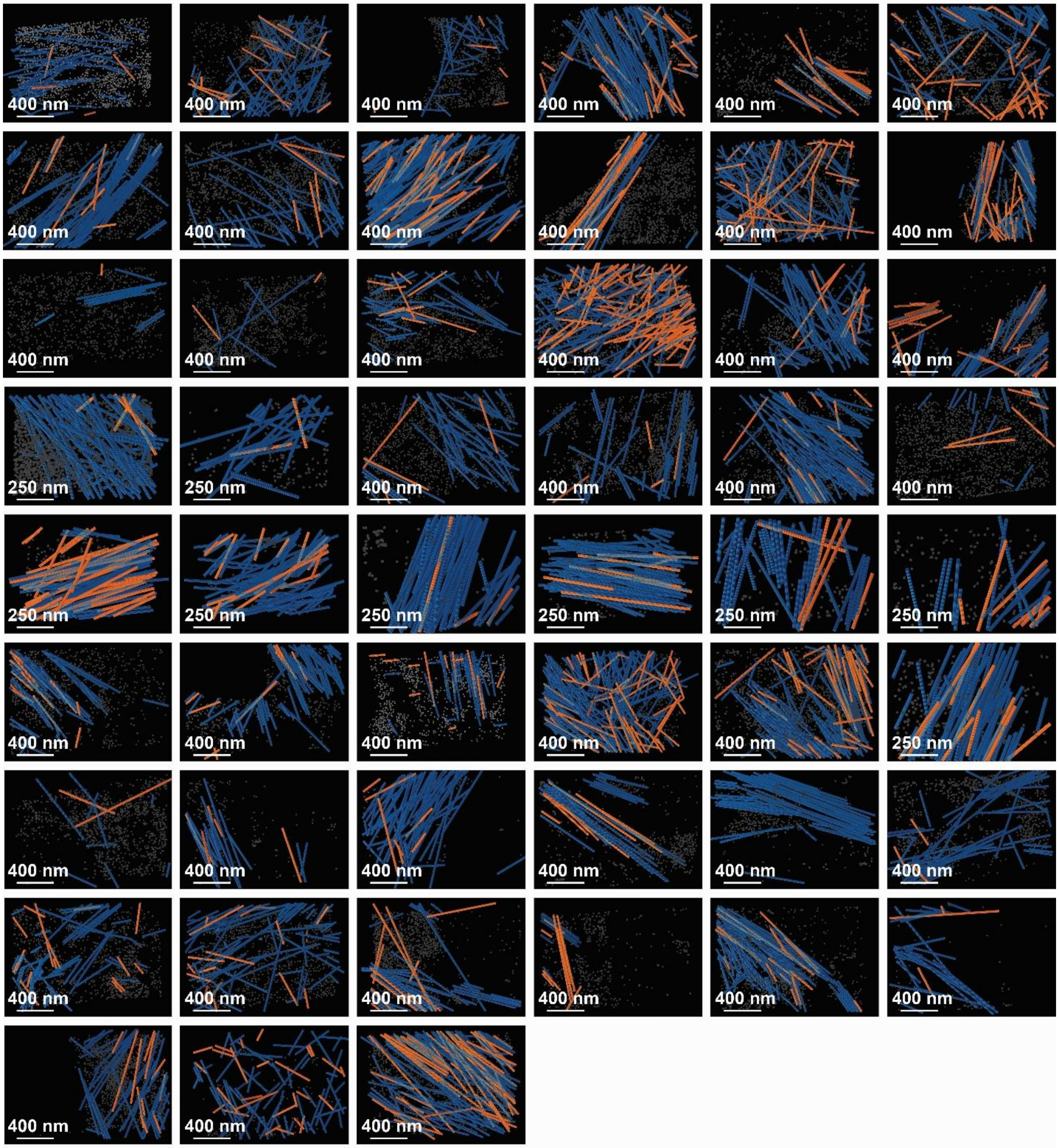

1002
1003 **Figure 3 – supplement 1: Tomographic reconstructions of Centrinone-treated motor-**
1004 **active monopolar spindles.** Blue/orange: microtubules colored by polarity. Gray: ribosomes.
1005

bioRxiv preprint doi: https://doi.org/10.64898/2026.01.16.699769; this version posted January 16, 2026. The copyright holder for this preprint (which was not certified by peer review) is the author/funder, who has granted bioRxiv a license to display the preprint in perpetuity. It is made available under aCC-BY 4.0 International license.

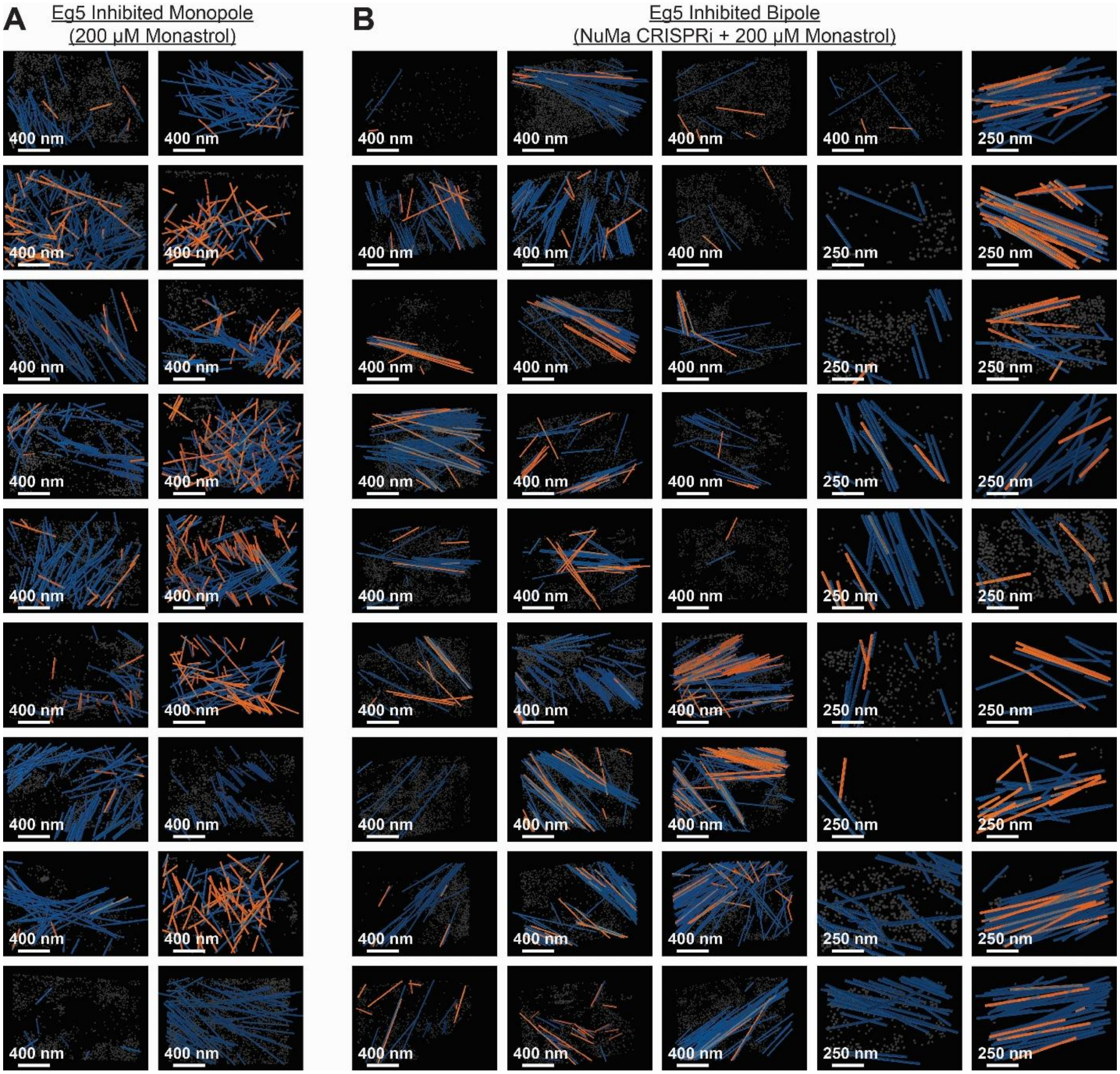

Figure 4 – supplement 1: Tomographic reconstructions of Eg5-inhibited monopolar and bipolar spindles. Blue/orange: microtubules colored by polarity. Gray: ribosomes. A) Eg5-inhibited monopolar spindles B) Eg-5 inhibited, NuMa CRISPRi DualKO bipolar spindles

bioRxiv preprint doi: https://doi.org/10.64898/2026.01.16.699769; this version posted January 16, 2026. The copyright holder for this preprint (which was not certified by peer review) is the author/funder, who has granted bioRxiv a license to display the preprint in perpetuity. It is made available under aCC-BY 4.0 International license.

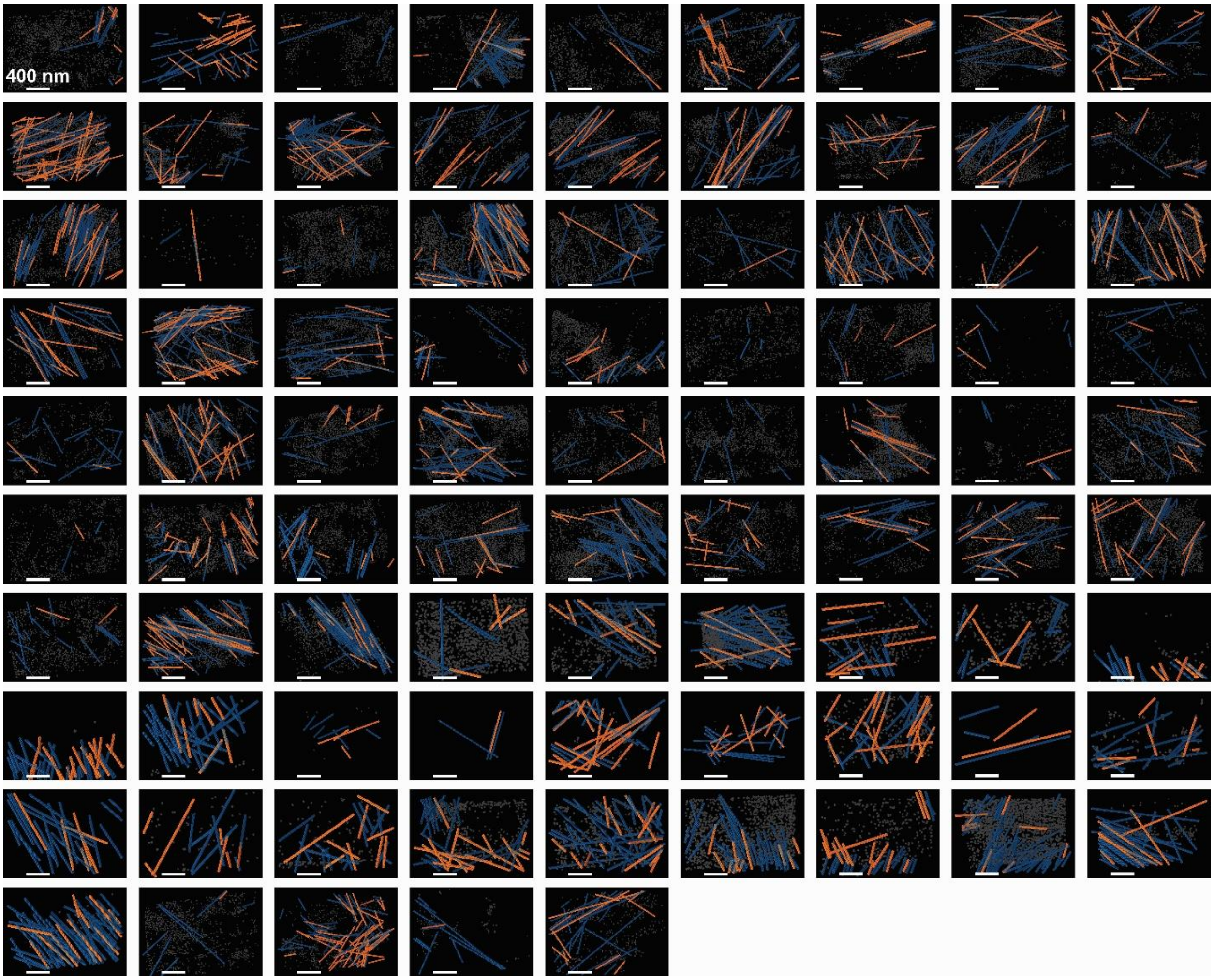

Figure 4 – supplement 2: Tomographic reconstructions of Eg5-active NuMa CRISPRi dynein-inhibited spindles. Blue/orange: microtubules colored by polarity. Gray: ribosomes. A) Eg5-inhibited monopolar spindles B) Eg-5 inhibited, NuMa CRISPRi DualKO bipolar spindles

bioRxiv preprint doi: https://doi.org/10.64898/2026.01.16.699769; this version posted January 16, 2026. The copyright holder for this preprint (which was not certified by peer review) is the author/funder, who has granted bioRxiv a license to display the preprint in perpetuity. It is made available under aCC-BY 4.0 International license.

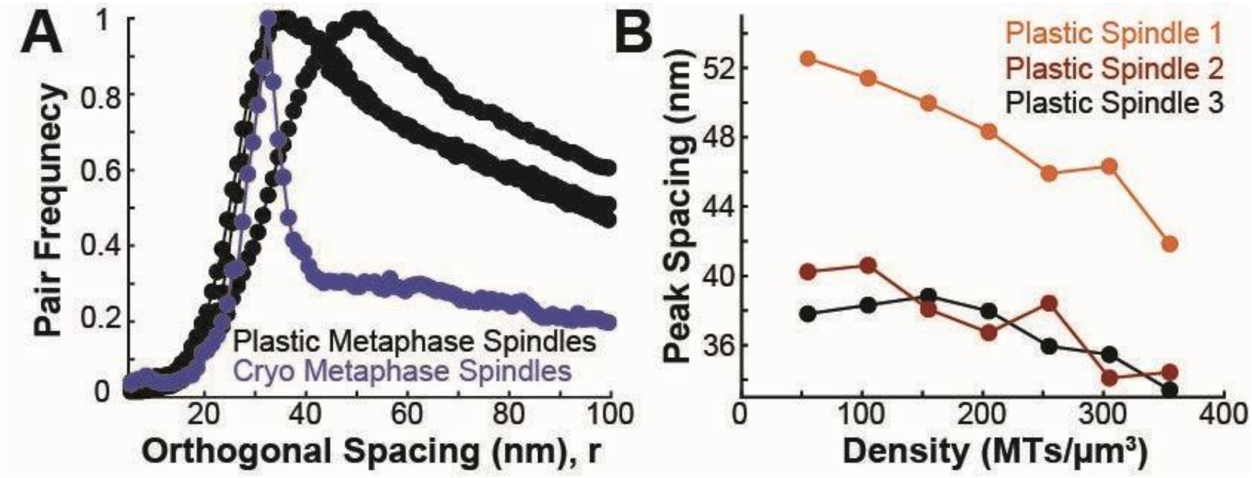

Figure 6 - supplement 1: Microtubule density sets spacing in each of the three plastic HeLa spindles. A) Frequency of spacing between microtubules in each of the three plastic spindle reconstructions (black) and cryotomograms (blue). B) Peak spacing between microtubules vs local density for each of the three plastic spindle reconstructions.
